# Perceptions and use of self-management support strategies to improve the management of spine pain patients in a French-Canadian chiropractic teaching program: a mixed method study

**DOI:** 10.1186/s12998-025-00611-1

**Published:** 2025-10-27

**Authors:** Philippe Rousseau, Danikel Giroux, Chloé Branconnier, Emile Marineau, Jocelyn J. Lemire, André Bussières

**Affiliations:** 1 Département d’Anatomie, Université du Québec À Trois-Rivières, Trois-Rivières, Canada; 2 Département Chiropratique, Université du Québec À Trois-Rivières, Trois-Rivières, Canada; 3 Groupe de Recherche Sur Les Affections Neuromusculosquelettique de L’Université du Québec À Trois-Rivières, Trois-Rivières, Canada

**Keywords:** Patient Activation, Evidence-Based Practice, Self-management, Theoretical Domain Framework, Chiropractic, Professional education

## Abstract

**Background:**

Clinical guidelines for managing non-specific spine pain recommend providing patient education and self-management support strategies (SMSS) as first-line treatment. However, SMSS implementation in daily chiropractic care remains challenging. This study aimed to assess the level of patient activation in their care, explore chiropractic senior interns and clinician supervisors’ beliefs about evidence-based practice (EBP) and self-management support, and identify theoretical barriers and facilitators to implementing SMSS.

**Methods:**

We used a three-phase mixed-methods convergent design. In phase 1, during the spring and summer of 2022, 250 consecutive adults with spine pain at the outpatient chiropractic clinic at the Université du Québec à Trois-Rivières, Quebec, Canada, were invited to complete the patients’ activation measure (PAM). In phase 2, all senior interns (n = 39) and clinician supervisors (n = 29) were invited to complete three self-administered online questionnaires: 1) EBP Beliefs and Implementation Scales, 2) Pain Attitudes and Beliefs Scale (PABS), and 3) the Practice Style questionnaire. In phase 3, patients, interns and clinicians having completed the questionnaires were convened to semi-structured individual interviews based on the Theoretical Domains Framework (TDF).

**Results:**

In phase 1, three quarters of patients (76.3%) reported a moderate-to-high level of activation. In phase 2, interns and clinician supervisors had similar EBP Beliefs mean scores (62.8% and 62.5%, respectively) and EBP Implementation scores (28.6% and 38.2%, respectively). For the PABS, no predominant biomedical or behavioural treatment orientations were observed among interns (mean (SD) = 34.8 (6.3) /60 vs 36.7 (3.5) /48) or clinicians (34.7 (9.1) /60 vs 34.6 (4.9) /48). Interns primarily had a pragmatic practice style, whereas clinicians were equally pragmatic and receptive. In phase 3, four key TDF domains emerged for patients (Social influences, Behavioural regulation, Emotions, and Goals); five for interns (Knowledge, Environmental Context and Resources, Skills, Memory, Attention and Decision Process, and Goals), and four for clinicians (Knowledge, Environmental Context and Resources, Social Influences and Beliefs on Consequences).

**Conclusion:**

Although patients demonstrated moderate-to-high activation, EBP and SMSS implementation among interns and supervisor was limited. Treatment orientation, practice style, and contextual factors highlight the need for targeted educational and organizational strategies to bridge the knowledge-practice gap.

**Supplementary Information:**

The online version contains supplementary material available at 10.1186/s12998-025-00611-1.

## Background

Musculoskeletal disorders (MSD) represent a leading cause of global disability [1–3], with approximately 1 billion people living with spine pain worldwide [4]. Chronic spine pain significantly impacts quality of life and is associated with reduced physical functioning, poor sleep quality, comorbidities such as mood disorders and cardiovascular conditions [5, 6], social isolation, and economic hardship [7, 8], and is a major contributor to job absenteeism and reduced productivity [9–11].

Patients with chronic low back pain and neck pain often consult primary health care providers, including chiropractors for pain relief. Clinical practice guidelines on the management of spine pain recommend that health care providers provide patient education and self-management support strategies (SMSS) as first-line treatment [12]. SMSS can be defined as “individual’s ability to manage their symptoms, the related physical and psychosocial consequences, and lifestyle changes inherent to living with a chronic condition” [13]. While SMSS are deemed useful to reduce pain, disability and psychological distress, health care providers, including chiropractors, do not routinely provide SMSS to their patients [14, 15].

Successful implementation of SMSS in daily patients’ care is challenging [16, 17]. Since it may be easier to influence habits while students undergo professional training rather than when they are already established in clinical practice, incorporating clinical practice guideline recommendations on SMSS within teaching curriculums of future musculoskeletal (MSK) health care providers is advisable [18]. A recent study conducted at the Canadian Memorial Chiropractic College (CMCC), Ontario, Canada, suggested that final year chiropractic interns and their clinician supervisors often omitted to deliver SMSS to patients [19]. Patients’ compliance to self-management was partly hindered by the lack of communication with their care provider [19].

To date, no studies have explored factors likely to hinder the utilization of SMSS in a university-based French chiropractic teaching program. Thus, the current study undertaken in the chiropractic teaching program at the Université du Québec à Trois-Rivières (UQTR), Quebec, Canada, aimed to 1) assess the level of patient activation in their own care, 2) explore chiropractic senior interns and their clinician supervisors’ beliefs about evidence-based practice (EBP) and self-management support, and 3) understand theoretical barriers and facilitators toward implementing SMSS with their patients.

## Methods/design

A three-phase mixed-methods convergent design was used. Mixed-methods research using quantitative and qualitative research integration can enhance contextual understanding of the complex, multi-level challenge of spine problems and care approach [20, 21]. The purpose was to examine the same phenomenon by interpreting qualitative and quantitative results, that is by bringing data analysis together at the interpretation stage [22]. Ethical approval was obtained on June 29th, 2022 from the Research Ethics Board of the Université du Québec à Trois-Rivières (CER 21–277-07.13; CER-22–290-08–01.11). Written informed consent was obtained from all participants.

### Context

This study took place at the Student Outpatient Chiropractic Clinic at UQTR, which holds the only French chiropractic teaching program in North America. Each year, 47 new students enter the five-year chiropractic program. During the last 18 months of their education, chiropractic interns deliver patient care under the supervision of one of 29 licensed clinician supervisors.

### PHASE 1. Estimating patient activation level (quantitative data)

#### Participants

During the spring and summer of 2022, we recruited consecutive adult patients (18–80 years old) undergoing chiropractic care for a complaint of non-specific neck, mid-back or low back pain. An upper age limit of 80 years was applied to minimize potential challenges related to cognitive decline, sensory impairment, or physical fatigue that could affect the accuracy and completeness of self-reported data. This limit also facilitated the use of study procedures such as online survey completion via QR code. Participants able to read and hold a conversation in French were eligible to participate.

#### Procedures

Patients were recruited through two methods. First, senior chiropractic interns identified eligible participants based on their clinical diagnostic. Second, a poster in the main corridor of the UQTR outpatient chiropractic clinic described the purpose of the study and included a QR code linking to additional information. The first 250 patients were invited by the senior chiropractic interns to complete a paper-based or an online version of the Patient Activation Measure (PAM) survey questionnaire [22] to assess their level of self-care activation after consenting to participate. The online version was developed on the UQTR in-house web-based survey tool (Banque Interactive de Questions-BIQ), with responses stored securely on the university server. Submission required clicking a ‘‘saved’’ button; however, 29 electronic questionnaires were not saved and were unusable for analysis. Of the remaining 221 questionnaires, 31 participants reported no spine pain and were excluded, leaving 190 valid questionnaires.

### Data collection

#### Study instrument

We used the French version of Patient Activation Measure (PAM), a widely used and validated tool designed to indicate the level of participation in SMS [23–25]. PAM is property of Insignia Health Group, and its use for research purpose includes the analysis of the PAM scores. The PAM questionnaire includes 13 general health items, with scores ranging between 0 and 100. The final score can be divided into four subgroups known as ‘levels of activation’, ranging from low (low score) to high activation level (high score) according to the respondent experience, observation, and other health-related behaviours (Table 1). Participants’ personal information (name, phone, email) was we collected to contact participants was replaced with unique ID numbers, and the master sheet stored securely in a locked filing cabinet in JJL’s office.

**Table 1:** Participants characteristics and patients PAM scores

Characteristics	Patients Mean (SD), N (%) (n = 190)	Interns Mean (SD), N (%) (n = 38)	Clinician supervisors Mean (SD), N (%) (n = 27)
Mean age (years) 20–30 years 31–40 years 41–50 years > 50 years	38.6 (17.7)	– 37 (97.4%) 1 (2.6%)	– – 7 (24.1%) 7 (24.1%) 13 (44.8%)
Gender (Females)	122 (62%)	26 (68.4%)	13 (44.8%)
Years of professional experience (mean)	–	1.5	23
Graduated from a Canadian chiropractic institution	–	–	24 (82%)
Highest level of education Doctorat premier cycle Master’s degree PhD Fellowship	–	–	12 9 2 5
Reported knowing about EBP	–	38 (97.4%)	28 (96.6%)
Acute pain (< 3 months)	79 (35.7%)	–	–
Chronic pain (> 3 months)	135 (64.2%)	–	–
PAM score Mean (Max = 100)	64.1 (12.0)	Description	
PAM Activation Levels: Level 1	17 (7.4%)	Lowest score—Individuals tend to be passive and feel overwhelmed by managing their own health. They may not understand their role in the care process	
Level 2	31 (16.3%)	Individuals may lack the knowledge and confidence to manage their health	
Level 3	95 (50.0%)	Individuals appear to be taking action but may still lack the confidence and skill to support their behaviours	
Level 4	50 (26.3%)	Highest score—Individuals have adopted many of the behaviours needed to support their health but may not be able to maintain them in the face of life stressors	

#### Sample size

To assess the patients’ participation in SMSS, the sample size was calculated using the following equation: N ≥ (1.96)^2^ * SD^2^ / d^2^ [26], using the standard deviation (SD) and margin of error (d) from PAM results in previous studies [19, 27, 28]. In total, 216 subjects were needed for this study (1.96^2^ * 15^2^) / 2^2^.

#### Data analysis

The total PAM scores (calibrated from score 0 = ‘No activation’ to 100 = ‘High activation’), and sub-scores (levels 1–4) were calculated by the Insignia Health Group [23]. An ordinal regression model was performed to identify clinical variables (spine pain region; number of body pain region affected; and duration of spine pain) associated with patient level of activation (PAM scores). We report McFadden’s pseudo-R-squares (R^2^), odds ratios (ORs), confidence intervals (CIs), and significance levels.

### PHASE 2. Exploring senior interns’ and clinician supervisors’ knowledge, attitude and behaviours

#### Participants

To be eligible, senior chiropractic interns had to be in their final year at the UQTR chiropractic program, and licenced chiropractors had to be actively working as clinician supervisors in the Student Outpatient Clinic in the spring of 2022.

#### Procedures

Invitations to participate in the study were sent to all eligible senior interns (n = 39) and clinician supervisors (n = 29) using the institutional emails. Interested participants accessed a link to online questionnaires after completing a consent form. We first collected sociodemographic data from the senior interns (age and gender) and clinician supervisors (age, gender, country of graduation, part- or full-time practitioners, number of years of experience, highest level of education). Participants were then asked to complete three self-administered questionnaires: 1) EBP Beliefs and Implementation Scales, 2) Pain Attitudes and Beliefs Scale (PABS), and 3) Practice Style questionnaire.

#### Evidence-based practice (EBP) beliefs and implementation scales

This questionnaire contains two sections. The EBP Beliefs Scale consists of 16 items to assess participants’ beliefs about the value of using EBP and their ability to use it. The EBP Implementation Scale is an 18-item questionnaire measuring the extend of use of EBP in someone’s practice. The EBP beliefs Scale is rated on a 5-point Likert scale (‘Strongly Agree’ = 1 to ‘Strongly Disagree’ = 5). The EBP Implementation Scale is rated on a 5-point scale, indicating how often the items were performed in the last 8 weeks, with 1 representing « zero times» and 5 representing « more than eight times». Both instruments have acceptable psychometric properties [29].

#### Pain attitudes and beliefs scale (PABS)

The PABS questionnaire assesses the strength of two treatment orientations of health care practitioners: biomedical and behavioural (i.e. psychosocial) orientations [30]. The amended version of the PABS is comprised of 19 items (10 biomedical and 9 behavioural items) [31]. Each question is rated on a 6-point scale “(‘Totally disagree’ = 1 to ‘Totally agree’ = 6)”, with higher scores on a subscale indicating a stronger treatment orientation [31]. The PABS also has acceptable psychometric properties [30, 32].

#### Practice style questionnaire

The Practice Style questionnaire is used to classify healthcare practitioners into 4 categories based on their style of practice (Seekers, Receptives, Traditionalists, and Pragmatists), according to responses obtained from a combination of three underlying factors: 1) extent to which scientific evidence, rather than clinical experience and authority, is perceived as the best source of knowledge; 2) degree of comfort with clinical practices that are out of step with the local community’s practices or the recommendations of leaders; and 3) importance associated with managing workload and patient flow while maintaining general patient satisfaction [33]. This questionnaire includes 17 statements about clinicians’ practice rated on 5-point Likert scale (‘Strongly Agree’ = 1 to ‘Strongly Disagree’ = 5).

#### Analysis

Descriptive analysis (means, standard deviations, count, percentages) was conducted using SPSS version 28 (IBM Corp, New York) [34]. The scores were calculated for each subscale of the EBP Beliefs and Implementation, PABS, and Practice style questionnaires. Comparison between means of EBP beliefs and implementation scale and PABS were calculated for interns and clinicians. Due to the small number of senior chiropractic interns in the final year of the program and eligible clinician supervisors, we could not explore associations between demographic variables and scores obtained in the three questionnaires using regression analyses.

### PHASE 3. Identifying theoretical barriers and enablers (qualitative data)

In the summer of 2022, we conducted online semi-structured individual interviews with spine pain patients, senior interns and clinician supervisors based on the Theoretical Domains Framework (TDF) [35] encompassing 14 domains: *Knowledge, Skills, Environmental Context & Resources, Emotion, Beliefs about Capabilities, Beliefs about Consequences*, *Memory, Attention and Decision making, Optimism, Reinforcement, Intention, Goals and Social Influence.*

#### Participants

Using a random number, up to 30 of the 190 spine pain patients returning a valid PAM questionnaire were selected, and 13 of them were invited by phone by one of the author (JL) to take part in 45–60-min individual interview. Data saturation was obtained for most of the TDF domains after the 11th interview, and for all domains after the last interview. Similarly, a convenient sample of 12 senior interns and 10 clinician supervisors who had completed all study questionnaires were invited to participate in semi-structured individual interview using participants’ institutional email. Ten to thirteen individuals are considered an appropriate sample size for TDF interview study [36]. Patients and senior interns agreeing to participate received a 50-dollar gift card for their time.

#### Data collection

Qualitative interviews aimed to identify barriers and facilitators likely to influence the use of self-management and related support strategies. We adapted English TDF interview topic guides from a similar project [19]. Two bilingual authors with clinical (JL) and knowledge transfer (AB) expertise translated the two interview guides into French, each made up of 25 open-ended questions, with 1 to 3 questions per domain. Questions for patients explored their beliefs about spine pain recovery, activity limitation, exercise, and physical activity. Questions for senior interns and clinician supervisors explored their views about patient self-management support strategies in the context of undergraduate health professional education. All participants completed and signed an informed consent form prior to the interview. Interviews lasted between 20 and 45 min and were conducted via the Zoom platform (Zoom Video Communication, Inc. Zoom Version: 5.11.9, San Jose, CA, USA) by team members (JL & AB). All interviews were anonymized and transcribed verbatim.

#### Data analysis

Qualitative data were analyzed using a thematic content analysis approach using similar strategies described in other studies [19, 37, 38]. Briefly, four assessors (CB, DG, JL, EM) independently coded transcripts using Dedoose software (Los Angeles, California, USA), and regularly met to compare coding and reach consensus. The senior author (AB) experienced in qualitative analysis using the TDF was consulted at each step, and differences in interpretation were reconciled. Specifically, transcripts were divided into different statements (unit of meaning) which were coded according to the relevant TDF domain. Similar statements within the domains were grouped into specific beliefs. A specific belief is defined as “a core statement that captures a common theme from multiple response statements and provides detail about the role of a given domain in influencing practice behaviour” [37, 39]. The specific beliefs were divided into three groups based on their likelihood to either increase (facilitator), decrease (barrier), or have no influence on the use of SMSS. Finally, we identified salient barriers based on three criteria applied concomitantly: frequency of belief, importance of the belief, and contrasting beliefs. Quotes reported in this paper were translated using the free machine translation service provided by Deepl.com (DeepL SE in Cologne, Germany), and reviewed for accuracy by the team.

## Results

### Phase 1. patients PAM questionnaire

Of the 250 patients completing the PAM, 190 provided valid questionnaires (76% response rate). The mean age was 38.8 years old (SD = 17.6; range = 18–80) and 62% of the respondents were females. The average PAM score was 64.1% (SD = 17.7) suggesting an overall moderate to high activation level (Table 1). Specifically, over one quarter of participants (26.3%) scored in the 4th (highest) activation level, half (50.7%) in the 3rd level of activation, and the remaining were in lower levels of activation with 16.3% in the 2nd level, and 7.4% in the first (lowest) level.

#### PAM statistical analyses

Thoracic pain is significantly associated with a lower probability of belonging to higher level of activation according to PAM score. Cervical and lumbar pain and chronicity have no significant association with the level of activation (Appendix 1, Table 1).

### Phase 2. EBP beliefs and implementation scales, PABS, and practice style questionnaire

In total, 38 out of 39 senior interns (97% response rate) and 27 out of 29 clinicians (93% response rate) completed the EBP Beliefs and Implementation Scales, the PABS, and the Practice Style questionnaires. Senior interns were mostly females (68%), while a small majority of clinician supervisors were males (55%), most of them having graduated from a Canadian teaching institution (n = 24; 82%), with a mean of 23 years of clinical experience. Nearly all interns (97.4%) and clinicians (96.6%) reported having prior knowledge of EBP (Table 1).

### EBP beliefs and implementation scales

#### EBP beliefs scale

Table 2 reports the mean score of the EBP Beliefs Scale questionnaire. For senior interns, the total mean score was 62.8% (SD = 6.4; range = 50–73). The belief that EBP results in the best clinical care for patients had the highest endorsement, while the belief that EBP needs much time had the lowest score with only 7.9% of senior interns agreeing or strongly agreeing. Similarly for clinician supervisors, the total score mean was 62.5% (SD = 7.1; ranges 44–72). Items 1 and 4 had the highest score (Belief that EBP results in the best clinical care for patients and that critical appraisal of evidence is essential in EBP process) with 96.3%, while the lowest score item was the belief that EBP is difficult (11.1%).

**Table 2:** Evidence-based Practice (EBP) Beliefs and Implementation Scales

EBP beliefs scale (Strongly agreed / agreed)			EBP implementation scale (Responded > 6 times)		
Items	Interns (n = 38)	Clinicians (n = 27)	Items	Interns (n = 38)	Clinicians (n = 27)
1. Belief that EBP results in the best clinical care for patients	100%	96.3%	1. Used evidence to change my clinical practice	50.0%	38.5%
2. Clear about the steps of EBP	84.2%	85.2%	2. Critically appraised evidence from a research study	73.0%	37.5%
3. Can implement EBP	92.1%	84.6%	3. Generated a PICO question about my clinical practice	73.7%	30.8%
4. Belief that critical appraisal of evidence is essential in EBP process	92.1%	96.3%	4. Informally discussed evidence from a research study with a colleague	47.4%	46.2%
5. Belief that evidence-based guidelines can enhance clinical care	89.5%	96.2%	5. Collected data on a patient problem	68.4%	60.0%
6. Belief that one can search the best evidence in a time-efficient way	84.2%	74.1%	6. Shared evidence from a study/ies in the form of a report or presentation to > 2 colleagues	21.1%	28.0%
7. Belief that one can overcome barriers to EBP implementation	81.6%	81.5%	7. Evaluated the outcomes of a practice change	10.5%	16.0%
8. Can implement EBP in a time-efficient way	76.3%	81.5%	8. Shared an EBP guideline with a colleague	23.7%	36.0%
9. Belief that implementing EBP will improve the care to patients	94.7%	92.6%	9. Shared evidence from a research study with a patient/family member	23.7%	32.0%
10. Ability to measure the outcomes of clinical care	92.1%	85.2%	10. Shared evidence from a research study with a multidisciplinary team member	21.6%	32.0%
11. Belief that EBP needs much time	7.9%	14.8%	11. Read and critically appraised a clinical research study	73.7%	40.0%
12. Can access the best resources to implement EBP	78.9%	70.4%	12. Accessed the Cochrane database of systematic reviews	28.9%	26.9%
13. Belief that EBP is difficult	28.9%	11.1%	13. Used an EBP guideline or systematic review to change clinical practice where I work	42.1%	40.0%
14. Know how to implement EBP sufficiently enough to make practice changes	71.1%	70.4%	14. Evaluated a care initiative by collecting patient outcome data	18.9%	26.9%
15. Confidence to implement EBP in work	78.4%	80.8%	15. Shared the outcome data collected with colleagues	21.6%	34.6%
16. Belief that the care delivered is evidence-based	92.1%	74.1%	16. Changed practice based on patient outcome data	34.2%	34.6%
			17. Promoted the use of EBP to my colleagues	23.7%	44.0%
Mean total score	62.8%	62.5%		38.6%	35.5%
*p*-value	0,367		*p*-value	0,299	

#### EBP implementation scale

The total mean score for senior interns was 38.6% (SD = 21.8; range = 10–68), and 35.5% (SD = 9.6 ranges = 0–68) for clinician supervisors, respectively (Table 2). The two items that had the highest number of senior interns, who responded more than 6 times, were items 3 and 11 with a response of 73.7% for each. These items represent that most senior interns have generated a search strategy using PICO question (Population, Intervention, Comparison, Outcome) about their clinical practice and read and critically appraised a clinical research study. For the clinician supervisors, 60% of them have collected data on a patient problem more than 6 times. While very few senior interns (10.8%) and clinician supervisors (16%) evaluated the outcomes of a practice change more than 6 times.

### Pain attitudes and beliefs scale (PABS)

Results from the PABS questionnaire among senior interns (mean (SD) = 34.8 (6.3) /60 vs 36.7 (3.5) /48) and supervisory clinicians (34.7 (9.1) /60 vs 34.6 (4.9) /48) revealed no significant difference in biomedical and behavioral treatment orientations (Table 3).

**Table 3:** Pain Attitudes and Beliefs Scale

Group	Biomedical treatment orientation Mean score/60 (SD)	Behavioural treatment orientation Mean score/48*(SD)	Mean (SD); p-value (2-tailed):
Intern (n = 33)	34.8 (6.3)	36.7 (3.5)	1.91 (7.52); *P* = 0,154
Clinicians (n = 22)	34.7 (9.1)	34.6 (5.0)	0.45 (10.5); *P* = 0,984

### Practice style questionnaire

**Fig. 1:**
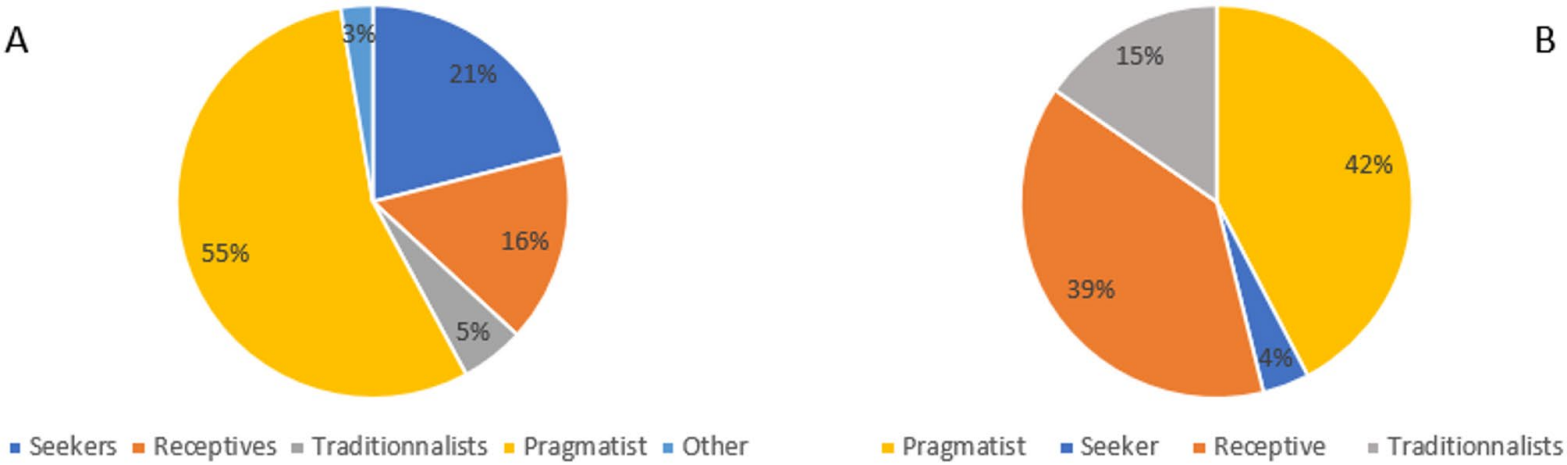
Practice Style Questionnaire results for (**A**) Interns and (**B**) Clinician supervisors

Senior interns tended to be pragmatic (55%), while clinician supervisors were almost equally receptive (39%) and pragmatic (42%), with a few very traditionalists (5%) or seekers (4%) (Fig. 1; Appendix 1, Table 2a and b).

### Phase 3. qualitative interviews

#### Spine pain patients

Among the 13 chronic spine pain patients we interviewed (mean age = 44.3; SD = 17.2 years, 69% female), we identified 122 specific beliefs and 40 themes from the 462 utterances across the 14 TDF domains (Appendix 1, Table 3). The following provides an overview of the four key TDF domains: *Social influences, Behavioural regulation, Emotions, and Goals.*

#### Social influences

Twenty-one utterances were summarized into six specific beliefs (5 facilitators; 1 barrier) forming one theme: senior interns and patients’ families had a positive impact on their use of SMSS. One participant mentioned: " All the feedback I’ve received, both from my intern and from my family, is to continue with the exercises. So everyone is kind of encouraging me in that. " (P1q44).

#### Behavioural regulation

Forty-two utterances were summarized into eight specific beliefs (5 facilitators; 3 barriers) and the three themes: Patients missing their appointments were the main barrier. In contrast, visual reminders were deemed helpful, and pain was considered a useful regulator in the monitoring of SMSS. This was highlighted by one participant: " [The use of SMS] depends on the pain intensity I think. If I am really in pain, I know what to do because I have exercises that helps. (P12q7).

#### Emotions

Twenty-five utterances were summarized into 11 specific beliefs (5 facilitators; 6 barriers) and three themes: SMSS is associated with a sense of well-being and pride for many, but can be a source of guilt, stress, and frustration. One participant said: " It’s a small source of stress or frustration when I don’t do it. " (P12q32).

#### Goals

Forty utterances were summarized into 13 specific beliefs (all facilitators) and the three following themes: Reducing pain, improving physical attributes, and taking responsibility for one’s own care. One participant mentioned: " I decided to take care of myself. That’s why self-management is important to me, so that I can work on myself. " (P5q29).

### Senior interns

The 12 senior interns (mean age: 24.8 years, 50% females) agreeing to be interviewed had 17 months of experience managing patients in the UQTR Student Outpatient Clinic. A total of 443 utterances were identified, grouped under 106 specific beliefs, and 38 themes (Appendix 1, Table 4). The five key TDF domains were: *Knowledge, Environmental context and resources, Skills, Memory, attention and decision process, and Goals.*

#### Knowledge

Forty-three utterances were summarized into six specific beliefs (6 facilitators; 6 barriers) and two themes: Variable levels of general knowledge, and how best to apply SMSS. One intern mentioned: " I personally don’t remember having a full course on [brief action planning], but maybe we’ve learned so much that I don’t remember, but it might be interesting to see it more in our program. " (I1q15).

#### Environmental context and resources

Sixty-five utterances were summarized into 11 specific beliefs (4 facilitators; 7 barriers) and four themes: While resources on SMSS are available, lack of time, clinician supervisors’ heterogeneity, and insufficient exposure on SMSS hinder its integration. One intern mentioned: " There are many [clinicians] who have a lot of clinical experience and have been at the clinic for years, but who might be a bit less receptive to change. In my opinion, several clinicians may also not be the most up-to-date in terms of evidence." (9Iq36).

#### Skills

Seventeen utterances were summarized into 10 specific beliefs (4 facilitators; 6 barriers) and two themes: Senior intern’s unequal skills when applying SMSS, and patient communication. One intern mentioned: " There were occasions when the patient told me about an objective, but we didn’t define it properly and didn’t reach it. My lack of competence in implementing this kind of approach was probably the problem." (I6q40).

#### Memory, attention and decision process

Twenty-five utterances were summarized into seven specific beliefs (2 facilitators; 4 barriers) and three themes: Share decision making with patients, deciding not to use SMSS on the first clinical encounter, and patients’ reluctance to managing their own condition. This was illustrated by one intern who said: " I would suggest [exercises] to everyone. Of course, not at the first appointment, because it’s very busy. " (I6q37).

#### Goals

Forty-three utterances were summarized into 6 specific beliefs (5 facilitators; 1 barrier) and three themes: Educating patients on the importance of self-care, providing timely SMSS to patients, and doing all they can to improve patient health using SMART goals (Specific, Measurable, Achievable, Relevant, and Time-Bound goals). One intern mentioned: "I feel capable of giving [SMSS]. It’s definitely something I want to do—provide self-management strategies." (I10q12).

### Clinician supervisors

Nine clinician supervisors (mean age = 54 years; SD = 8 years, 33% female) agreed to participate. A total of 329 utterances were identified, grouped under 151 specific beliefs and 50 themes (Appendix 1, Table 5). The four key TDF domains were: *Knowledge, Environmental context and resources, Social Influences, and Beliefs about consequences.*

#### Knowledge

Forty-two utterances were summarized to 20 specific beliefs (12 facilitators, 8 barriers) and four themes: Training and access to scientific literature on SMSS would facilitate their use, senior interns have sufficient knowledge, clinician supervisors varying levels of knowledge and lack of training in SMSS are limiting factors, and written recommendations and audiovisual demonstrating exercises increase patient’s adherence. One clinician supervisor mentioned: " So, if there is training or resources put in place to help us [with SMSS], I think it will greatly help in the implementation. " (C4q29).

#### Social influences

Seventeen utterances were summarized to 13 specific beliefs (7 facilitators, 6 barriers) and the following two themes: patients, senior interns, clinician supervisors, and other professionals are in favour of the use of SMSS, the clinic administration should support the clinician’s use of SMSS. One clinician supervisor mentioned: "At the same time, I think it gives patients, well, us, our profession, a certain credibility, because we want to do everything we can to help the person with their condition as much as possible." (C4q13).

#### Environmental context and resources

Fifty-one utterances were summarized to 22 specific beliefs (6 facilitators, 16 barriers) and the following six themes: Lack of time, manpower (low ratio of clinician to interns), clinical support tools (e.g., vignettes, algorithms), training/workshops, and resource persons; not all clinicians effectively use available resources. One clinician supervisor indicated: " I also believe that using different modes of presentation, i.e. above all a visual support, either pre-recorded vignettes or examples of helping relationships, will simply facilitate the integration of the ability to carry out these processes. " (C2q38).

#### Beliefs about consequences

Nineteen utterances were summarized to seven specific beliefs (5 facilitators, 2 barriers) and two themes: Offering SMSS will help patients manage their condition successfully; SMSS is a way of empowering patients. One clinician supervisor said: "If you follow him and then guide him towards chronicity, it’s like not giving him the tools to help himself and then knowing that the patterns will come back, the pain will come back. " (3Cq20).

## Discussion

Although effective for reducing spine pain and disability [15, 38, 40], and improving the quality of life of related comorbidities [41, 42], SMSS is generally not well understood or utilized by healthcare professionals [43, 44]. This study aimed to assess patients’ involvement in their care, explore chiropractic senior interns and clinician supervisors’ beliefs about self-management support strategies (SMSS) and the use of EBP, and identify factors likely to hinder the implementation of SMSS in an undergraduate chiropractic program.

Together, our findings suggest that better results would be achieved if educational curriculums provided student learners and clinician supervisors with formal training on SMSS, easy-to-use tools such as the Brief Action Planning [45], while allocating sufficient time to utilize SMSS with chronic spine pain patients.

In patients with chronic pain, higher levels of activation are correlated with greater clinical improvement [46, 47]. Together, our finding and those from a related study conducted in an English chiropractic teaching institution in Ontario [19] indicate that people seeking care in chiropractic teaching clinics exhibit a higher degree of engagement in their care. Furthermore, adults experiencing disabling comorbidities and back-related restricted-activity days demonstrate a higher propensity to utilize medical care over chiropractic interventions. This suggests that patients who could potentially benefit from rehabilitative services may not be accessing these treatments adequately [48].

Approximately one third of individuals who experience an episode of back pain are at risk of recurrence within the first year [49]. Clinical guidelines for the management of back and neck pain emphasize the importance of avoiding prolonged bed rest, remaining physical activity, participating in home exercise programs, and returning to work promptly [50–53]. Our exploratory analysis suggests that patients with spinal pain, particularly those with thoracic pain or multiple site pain, tend to have a lower engagement in their care. Providing patients with educational resources and exercise programs as preventive strategies to mitigate the future impact of low back pain should be encouraged [54].

Consistent with findings in nursing education [55–57], senior interns in our study valued EBP but did not routinely apply, indicating a persistent gap between knowledge and implementation. Their EBP Beliefs and Implementation scores did not differ significantly from those of clinician supervisors, likely reflecting the influence of supervisors’ beliefs on students [58]. Lower scores on specific items may point to a disconnect between faculty delivering the academic curriculum (years 1–4), revised in 2008 to emphasize EBP, and clinicians in the UQTR outpatient clinic (years 4–5) who maintain active practice. Supervisors with lower EBP beliefs and implementation may be less likely to model or promote its use encourage during clinical placements.

A behavioural treatment orientation is generally associated with improve patients’ health outcomes [59–62]. In contrast to a related study in Ontario where CMCC senior interns exhibited a stronger behavioural than biomedical treatment orientation [44], neither interns nor supervisory clinician in our study showed a predominant treatment orientation. This may reflect an integrative approach to pain management, with orientation shifting according to pain type or patient needs. Alternatively, participants may hold more neutral or uncertain treatment beliefs.

As in studies of occupational therapists, physical therapists, physicians, and chiropractors, [33, 44, 63, 64], few of our study participants were “seekers” (i.e., clinicians whose decisions are driven primarily by scientific evidence). Most interns and many clinicians were “pragmatists,” prioritizing interventions perceived to yield quick results over those with strong empirical support, potentially explaining the mismatch between moderate EBP beliefs and low implementation scores [33]. Clinician supervisors displayed a more balanced distribution between pragmatic and receptive styles, suggesting some openness to integrating credible scientific and clinical sources alongside practical decision-making [33]. However, this receptiveness did not translate into higher EBP Implementation scores. This discrepancy may indicate that while supervisors are open to evidence in principle, structural constraints (e.g., time, resources), entrenched habits, or selective application of evidence limit consistent implementation. Alternatively, social desirability bias may have inflated EBP Beliefs scores, with respondents providing answers they perceived as professionally appropriate, despite lower reported engagement in EBP-related behaviours.

Overall, our findings suggest that while interns and clinician supervisors valued EBP conceptually, treatment orientation and practice style may limit its application in practice. Targeted educational initiatives and support for clinician supervisors could strengthen EBP integration of EBP in chiropractic training and clinical care.

Patients reported lacking time and confidence in their capabilities were barriers to adhering to clinicians’ advice on self-management. Capability and opportunity are two prerequisites for a patient to adhere to clinical recommendations and maintain behaviour [65]. Capability includes, but is not limited to patients’ education, helping identify situations where patients could relapse and equipping them with problem-solving skills. Opportunity can be achieved by giving feedback, having discussion, and giving support to patients to increase their adherence to self-management [66]. Thus, helping patients manage their time more efficiently, reflecting on their current behaviours and offering concrete examples, setting goals, and ensuring proper follow-ups to meet their goals may increase patient’s motivations and help develop their ability to self-management [66, 67].

While some senior interns felt confident about their ability to provide SMSS to their patients and had good intentions to do so, many felt overwhelmed during the initial and follow-up clinical encounters with all they need to accomplish, such as reports of findings and delivery of manual therapy. Senior interns also reported not formally learning about SMSS during their clinical training. Low levels of self-competency, knowledge and experiences can increase students’ levels of anxiety [68]. Improving students’ knowledge and skills and thus increasing their metacognitive competence can help them recognize the limitations of their abilities [69, 70]. Introducing clinical skills within a system-oriented problem-based curriculum early in the training curriculum may increase student confidence when dealing with real patients, consolidate theoretical knowledge and clinical skills, motivate learning, and increase students’ enthusiasm to become health care providers [71]. Senior interns also highlighted a lack of consistency among clinician supervisors regarding the use of SMSS, possibly due to different practice styles, varying educational background and/or range of clinical experiences [72].

Clinician supervisors generally believed that SMSS is essential to improve MSK conditions and felt confident about their ability to teach SMSS to senior interns. However, most reported lacking the time due to administrative duties such as frequent student evaluation and chart audits. As for dentistry [73, 74], clinician supervisors stressed the need of a clinician-to-student supervision ratio of 1:4, instead of the current 1:8 ratio, to allow prioritizing teaching SMSS to senior interns. Finally, clinician supervisors welcomed additional training to help standardize the teaching of SMSS.

**Call out box:** Summary of recommendations to facilitate the use of SMSS.

**Table:** 

1. Providing student learners and clinician supervisors with formal training on SMSS.	
2. Allocating sufficient time to utilize SMSS with chronic spine pain patients.	
3. Providing patients with educational resources and exercise programs as preventive strategies to mitigate the future impact of low back pain should be encouraged.	
4. Targeted educational initiatives and support for clinician supervisors to strengthen EBP integration of EBP in chiropractic training and clinical care.	
5. Helping patients manage their time more efficiently, reflecting on their current behaviours and offering concrete examples, setting goals, and ensuring proper follow-ups to meet their goals may increase patient’s motivations and help develop their ability to self-management.	
6. Improving students’ knowledge and skills and thus increasing their metacognitive competence can help them recognize the limitations of their abilities.	
7. Introducing clinical skills within a system-oriented problem-based curriculum early in the training curriculum may increase student confidence when dealing with real patients, consolidate theoretical knowledge and clinical skills, motivate learning, and increase students’ enthusiasm to become health care providers.	
8. Offer additional training to clinician supervisors to help standardize the teaching of SMSS.	
9. Reduce the ratio of clinician supervisor to student from 1:8 to 1:4 to allow prioritizing teaching SMSS to senior interns.	

### Future research

Investigating the adoption and use of EBP and SMSS throughout the entire undergraduate chiropractic teaching program may help capture how student beliefs, knowledge, and skills evolve as learners transition toward professional practice, thus help identify more effective teaching methods to link theoretical and practical learning. Knowledge gained in this study can inform the tailoring of knowledge translation interventions to increase the uptake and application of SMSS in chiropractic teaching institutions [19].

### Study limitations

Selection bias may have occurred as participation was on a voluntary basis. The small number of participants prevented us from conducting a multiple regression analysis to explore associations between demographic variables self-administered questionnaires, and also limits the generalizability of our results. Results of the Pain Attitude and Beliefs questionnaire should be interpreted with caution as one question from the behavioural subscale was removed due to inaccurate translation, possibly impacting validation. Lastly, the analysis of the qualitative interviews is subject to team members shared understanding of the definitions of the TDF domains and constructs, which may contribute to differences in participants’ interpretation.

## Conclusions

Participants seeking care for spine pain reported a moderate to high levels of engagement in their care. While theoretical knowledge and beliefs about EBP and SMSS were generally favorable, their consistent application in practice was suboptimal. These findings point to the importance of integrating SMSS and EBP training more systematically across all stages of the chiropractic curriculum and ensuring alignment between academic instruction and clinical supervision. Prioritizing and modelling these approaches in clinical placements could help bridge the gap between knowledge and practice, ultimately enhancing the quality and outcomes of spine care.

## Supplementary Information

Below is the link to the electronic supplementary material.

Supplementary Material 1

## Data Availability

The datasets used/or analyzed during the current study are available from the corresponding author on a reasonable request.

## Abbreviation

SMSS: Self-Management Support Strategies
EBP: Evidence-based practice
PAM: Patients’ Activation Measure
TDF: Theoretical Domains Framework
MSD: Musculoskeletal Disorder
MSK: Musculoskeletal
CMCC: Canadian Memorial Chiropractic College
UQTR: Université du Québec à Trois-Rivières
SD: Standard Deviation
R^2^: Pseudo R squared
ORs: Odd ratios
Cis: Confidence Intervals
PABS: Pain Attitudes and Beliefs Scale
PICO: Population, Intervention, Comparison, Outcome search strategy
SMART: Specific, Measurable, Achievable, Relevant, and Time-Bound goals

## References

[1] Williams A, et al. Musculoskeletal conditions may increase the risk of chronic disease: a systematic review and meta-analysis of cohort studies. BMC Med. 2018;16(1):167. 30249247 10.1186/s12916-018-1151-2 PMC6154805

[2] Wu A, et al. Global low back pain prevalence and years lived with disability from 1990 to 2017: estimates from the global burden of disease study 2017. Ann Transl Med. 2020;8(6):299. 32355743 10.21037/atm.2020.02.175 PMC7186678

[3] *Global burden of 369 diseases and injuries in 204 countries and territories, 1990–2019: a systematic analysis for the Global Burden of Disease Study 2019.* Lancet, 2020. **396**(10258): 1204–1222. 10.1016/S0140-6736(20)30925-9 PMC7567026 33069326

[4] Cieza A, et al. Global estimates of the need for rehabilitation based on the Global Burden of Disease study 2019: a systematic analysis for the Global Burden of Disease Study 2019. Lancet. 2020;396(10267):2006–17. 33275908 10.1016/S0140-6736(20)32340-0 PMC7811204

[5] de Luca KE, et al. The relationship between spinal pain and comorbidity: a cross-sectional analysis of 579 community-dwelling, older Australian women. J Manipulative Physiol Ther. 2017;40(7):459–66. 29037787 10.1016/j.jmpt.2017.06.004

[6] de Luca K, et al. Spinal pain, chronic health conditions and health behaviors: data from the 2016–2018 national health interview survey. Int J Environ Res Public Health. 2023. 10.3390/ijerph20075369. 37047983 10.3390/ijerph20075369 PMC10094294

[7] Maher C, Underwood M, Buchbinder R. Non-specific low back pain. Lancet. 2017;389(10070):736–47. 27745712 10.1016/S0140-6736(16)30970-9

[8] Chou R. Low back pain. Ann Intern Med. 2021;174(8):ITC113–28. 34370518 10.7326/AITC202108170

[9] Initiative, U.S.B.a.J. *THE HIDDEN IMPACT of Musculoskeletal Disorders on AMERICANS*. 2021 [cited 2024 May]; Available from: hrome-extension://efaidnbmnnnibpcajpcglclefindmkaj/https://www.boneandjointburden.org/docs/BMUS%20Impact%20of%20MSK%20on%20Americans%20booklet_4th%20Edition%20%282018%29.pdf.

[10] Yong RJ, Mullins PM, Bhattacharyya N. Prevalence of chronic pain among adults in the United States. Pain. 2022;163(2):e328–32. 33990113 10.1097/j.pain.0000000000002291

[11] *World Health Organization: Low back pain*. [cited 2024 May]; Available from: https://www.who.int/news-room/fact-sheets/detail/low-back-pain.

[12] Foster NE, et al. Prevention and treatment of low back pain: evidence, challenges, and promising directions. Lancet. 2018;391(10137):2368–83. 29573872 10.1016/S0140-6736(18)30489-6

[13] Mayo, N.E., *ISOQOL Dictionary of Quality of Life and Health Outcomes Measurement*. 2015: Isoqol.

[14] Newman S, Steed L, Mulligan K. Self-management interventions for chronic illness. Lancet. 2004;364(9444):1523–37. 15500899 10.1016/S0140-6736(04)17277-2

[15] Oliveira VC, et al. Effectiveness of self-management of low back pain: systematic review with meta-analysis. Arthritis Care Res (Hoboken). 2012;64(11):1739–48. 22623349 10.1002/acr.21737

[16] Straus, S., et al., *Knowledge Translation in Health Care: Moving from Evidence to Practice.* CMAJ : Canadian Medical Association journal = journal de l’Association medicale canadienne, 2010. **182**: p. E94–8. 10.1503/cmaj.081335 PMC2817344 20083566

[17] Bussières AE, et al. Evidence-based practice, research utilization, and knowledge translation in chiropractic: a scoping review. BMC Complement Altern Med. 2016;16:216. 27412625 10.1186/s12906-016-1175-0 PMC4944433

[18] Wood W, Rünger D. Psychology of habit. Annu Rev Psychol. 2016;67:289–314. 26361052 10.1146/annurev-psych-122414-033417

[19] Eilayyan O, et al. Promoting the use of self-management in patients with spine pain managed by chiropractors and chiropractic interns: barriers and design of a theory-based knowledge translation intervention. Chiropr Man Therap. 2019;27:44. 31636895 10.1186/s12998-019-0267-6 PMC6794734

[20] Creswell, J.W. and J.D. Creswell, *Research design: Qualitative, quantitative, and mixed methods approaches*. 2017: Sage publications.

[21] Green CA, et al. Approaches to mixed methods dissemination and implementation research: methods, strengths, caveats, and opportunities. Adm Policy Ment Health. 2015;42(5):508–23. 24722814 10.1007/s10488-014-0552-6 PMC4363010

[22] Creswell, J.W. and V.L.P. Clark, *Designing and Conducting Mixed Methods Research*. 2011: SAGE Publications.

[23] Hibbard, J.H. and H. Gilburt. *Supporting people to manage their health An introduction to patient activation*. 2014.

[24] Hibbard JH, et al. Development and testing of a short form of the patient activation measure. Health Serv Res. 2005;40(6 Pt 1):1918–30. 16336556 10.1111/j.1475-6773.2005.00438.x PMC1361231

[25] Rademakers J, et al. Clinicians’ beliefs and attitudes toward patient self-management in the Netherlands; translation and testing of the American Clinician Support for Patient Activation Measure (CS-PAM). BMC Health Serv Res. 2015;15:138. 25889832 10.1186/s12913-015-0799-y PMC4419501

[26] Charan J, Biswas T. How to calculate sample size for different study designs in medical research? Indian J Psychol Med. 2013;35(2):121–6. 24049221 10.4103/0253-7176.116232 PMC3775042

[27] Skolasky RL, et al. Psychometric properties of the patient activation measure among individuals presenting for elective lumbar spine surgery. Qual Life Res. 2009;18(10):1357–66. 19916057 10.1007/s11136-009-9549-0 PMC3561629

[28] Anderson JK, Wallace LM. Evaluation of uptake and effect on patient-reported outcomes of a clinician and patient co-led chronic musculoskeletal pain self-management programme provided by the UK National Health Service. Br J Pain. 2018;12(2):104–12. 29796262 10.1177/2049463717734015 PMC5958511

[29] Melnyk BM, Fineout-Overholt E, Mays MZ. The evidence-based practice beliefs and implementation scales: psychometric properties of two new instruments. Worldviews Evid Based Nurs. 2008;5(4):208–16. 19076922 10.1111/j.1741-6787.2008.00126.x

[30] Bishop A. Pain Attitudes and Beliefs Scale (PABS). J Physiother. 2010;56(4):279. 21213945 10.1016/s1836-9553(10)70014-x

[31] Houben RM, et al. Health care providers’ orientations towards common low back pain predict perceived harmfulness of physical activities and recommendations regarding return to normal activity. Eur J Pain. 2005;9(2):173–83. 15737810 10.1016/j.ejpain.2004.05.002

[32] Mutsaers JH, et al. Psychometric properties of the pain attitudes and beliefs scale for physiotherapists: a systematic review. Man Ther. 2012;17(3):213–8. 22277324 10.1016/j.math.2011.12.010

[33] Wyszewianski L, Green LA. Strategies for changing clinicians’ practice patterns. A new perspective. J Fam Pract. 2000;49(5):461–4. 10836780

[34] Corp, I., *IBM SPSS Statistics for Windows [Internet].*

[35] Cane J, O’Connor D, Michie S. Validation of the theoretical domains framework for use in behaviour change and implementation research. Implement Sci. 2012;7(1):37. 22530986 10.1186/1748-5908-7-37 PMC3483008

[36] Francis JJ, et al. What is an adequate sample size? Operationalising data saturation for theory-based interview studies. Psychol Health. 2010;25(10):1229–45. 20204937 10.1080/08870440903194015

[37] Francis JJ, et al. Evidence-based selection of theories for designing behaviour change interventions: using methods based on theoretical construct domains to understand clinicians’ blood transfusion behaviour. Br J Health Psychol. 2009;14(Pt 4):625–46. 19159506 10.1348/135910708X397025

[38] Du S, et al. Self-management program for chronic low back pain: a systematic review and meta-analysis. Patient Educ Couns. 2017;100(1):37–49. 27554077 10.1016/j.pec.2016.07.029

[39] Bussières AE, et al. Fast tracking the design of theory-based KT interventions through a consensus process. Implement Sci. 2015;10(1):18. 25880218 10.1186/s13012-015-0213-5 PMC4330935

[40] Hayden, J.A., et al., *Exercise therapy for chronic low back pain.* Cochrane Database Syst Rev, 2021. **9**(9): p. Cd009790. 10.1002/14651858.CD009790.pub2 PMC8477273 34580864

[41] Wagner EH, et al. Quality improvement in chronic illness care: a collaborative approach. Jt Comm J Qual Improv. 2001;27(2):63–80. 11221012 10.1016/s1070-3241(01)27007-2

[42] Allegrante JP, Wells MT, Peterson JC. Interventions to support behavioral self-management of chronic diseases. Annu Rev Public Health. 2019;40:127–46. 30601717 10.1146/annurev-publhealth-040218-044008 PMC6684026

[43] McGowan P. The challenge of integrating self-management support into clinical settings. Can J Diabetes. 2013;37(1):45–50. 24070748 10.1016/j.jcjd.2013.01.004

[44] Eilayyan O, et al. Promoting the use of self-management in novice chiropractors treating individuals with spine pain: the design of a theory-based knowledge translation intervention. BMC Musculoskelet Disord. 2018;19(1):328. 30205825 10.1186/s12891-018-2241-1 PMC6134709

[45] Gutnick D, et al. Brief action planning to facilitate behavior change and support patient self-management. Journal of Clinical Outcomes Management. 2014;21:17–29.

[46] Yao F, et al. Patient activation level and its associated factors in adults with chronic pain: a cross-sectional survey. Medicine (Baltimore). 2021;100(19):e25929. 34106661 10.1097/MD.0000000000025929 PMC8133271

[47] Greene J, Hibbard JH. Why does patient activation matter? An examination of the relationships between patient activation and health-related outcomes. J Gen Intern Med. 2012;27(5):520–6. 22127797 10.1007/s11606-011-1931-2 PMC3326094

[48] Chevan J, Riddle DL. Factors associated with care seeking from physicians, physical therapists, or chiropractors by persons with spinal pain: a population-based study. J Orthop Sports Phys Ther. 2011;41(7):467–76. 21654096 10.2519/jospt.2011.3637

[49] da Silva T, et al. Risk of recurrence of low back pain: a systematic review. J Orthop Sports Phys Ther. 2017;47(5):305–13. 28355981 10.2519/jospt.2017.7415

[50] Zhou T, Salman D, McGregor AH. Recent clinical practice guidelines for the management of low back pain: a global comparison. BMC Musculoskelet Disord. 2024;25(1):344. 38693474 10.1186/s12891-024-07468-0 PMC11061926

[51] Blanpied PR, et al. Neck pain: revision 2017. J Orthop Sports Phys Ther. 2017;47(7):A1-a83. 28666405 10.2519/jospt.2017.0302

[52] Bussières AE, et al. The Treatment of Neck Pain-Associated Disorders and Whiplash-Associated Disorders: A Clinical Practice Guideline. J Manipulative Physiol Ther. 2016;39(8):523-564.e27. 27836071 10.1016/j.jmpt.2016.08.007

[53] Organization WH. WHO guideline for non-surgical management of chronic primary low back pain in adults in primary and community care settings. Geneva: World Health Organization; 2023. 38198579

[54] de Campos TF, et al. Prevention strategies to reduce future impact of low back pain: a systematic review and meta-analysis. Br J Sports Med. 2021;55(9):468–76. 32646887 10.1136/bjsports-2019-101436

[55] Cruz JP, et al. Evidence-based practice beliefs and implementation among the nursing bridge program students of a Saudi University. Int J Health Sci (Qassim). 2016;10(3):405–14. 27610064 PMC5003584

[56] Lam CK, Schubert C. Evidence-based practice competence in nursing students: an exploratory study with important implications for educators. Worldviews Evid Based Nurs. 2019;16(2):161–8. 30977591 10.1111/wvn.12357

[57] Abu-Baker NN, et al. Evidence-based practice beliefs and implementations: a cross-sectional study among undergraduate nursing students. BMC Nurs. 2021;20(1):13. 33413336 10.1186/s12912-020-00522-x PMC7791790

[58] Norton L, et al. Teachers’ beliefs and intentions concerning teaching in higher education. High Educ. 2005;50:537–71.

[59] Hall A, et al. Physiotherapist-delivered cognitive-behavioural interventions are effective for low back pain, but can they be replicated in clinical practice? A systematic review. Disabil Rehabil. 2018;40(1):1–9. 27871193 10.1080/09638288.2016.1236155

[60] Richmond H, et al. The effectiveness of cognitive behavioural treatment for non-specific low back pain: a systematic review and meta-analysis. PLoS ONE. 2015;10(8):e0134192. 26244668 10.1371/journal.pone.0134192 PMC4526658

[61] van Erp RMA, et al. Effectiveness of primary care interventions using a biopsychosocial approach in chronic low back pain: a systematic review. Pain Pract. 2019;19(2):224–41. 30290052 10.1111/papr.12735 PMC7379915

[62] Williams AC, Eccleston C, Morley S. Psychological therapies for the management of chronic pain (excluding headache) in adults. Cochrane Database Syst Rev. 2012;11(11):Cd007407. 23152245 10.1002/14651858.CD007407.pub3 PMC6483325

[63] Korner-Bitensky N, et al. Practice style traits: do they help explain practice behaviours of stroke rehabilitation professionals? J Rehabil Med. 2007;39(9):685–92. 17999005 10.2340/16501977-0106

[64] Green LA, Gorenflo DW, Wyszewianski L. Validating an instrument for selecting interventions to change physician practice patterns: a Michigan Consortium for Family Practice Research study. J Fam Pract. 2002;51(11):938–42. 12485547

[65] Michie S, van Stralen MM, West R. The behaviour change wheel: a new method for characterising and designing behaviour change interventions. Implement Sci. 2011;6:42. 21513547 10.1186/1748-5908-6-42 PMC3096582

[66] Söderlund A, von Heideken Wågert P. Adherence to and the maintenance of self-management behaviour in older people with musculoskeletal pain-a scoping review and theoretical models. J Clin Med. 2021. 10.3390/jcm10020303. 33467552 10.3390/jcm10020303 PMC7830780

[67] Lenzen SA, et al. Disentangling self-management goal setting and action planning: a scoping review. PLoS ONE. 2017;12(11):e0188822. 29176800 10.1371/journal.pone.0188822 PMC5703565

[68] Sarikaya O, Civaner M, Kalaca S. The anxieties of medical students related to clinical training. Int J Clin Pract. 2006;60(11):1414–8. 16787438 10.1111/j.1742-1241.2006.00869.x

[69] Mavis B. Self-efficacy and OSCE performance among second year medical students. Adv Health Sci Educ Theory Pract. 2001;6(2):93–102. 11435761 10.1023/a:1011404132508

[70] Kruger J, Dunning D. Unskilled and unaware of it: how difficulties in recognizing one’s own incompetence lead to inflated self-assessments. J Pers Soc Psychol. 1999;77(6):1121–34. 10626367 10.1037//0022-3514.77.6.1121

[71] Khalil MS, et al. Students, faculty perceptions and effectiveness of the early introduction of clinical skills teaching in the medical curriculum. J Taibah Univ Med Sci. 2023;18(2):310–20. 37102079 10.1016/j.jtumed.2022.09.008 PMC10124110

[72] Dubuc É, et al. Chiropractic techniques and treatment modalities included in academic programs: a survey of chiropractic educational institutions. J Chiropr Educ. 2022;36(2):84–92. 35481855 10.7899/JCE-21-32 PMC9536225

[73] Waterhouse P, et al. The development of a primary dental care outreach course. Eur J Dent Educ. 2008;12(1):8–16. 18257759 10.1111/j.1600-0579.2007.00464.x

[74] Snider KT, et al. Trainer-to-student ratios for teaching psychomotor skills in health care fields, as applied to osteopathic manipulative medicine. J Am Osteopath Assoc. 2012;112(4):182–7. 22522517

