# Supplementary material for: Perceptions and use of self-management support strategies to improve the management of spine pain patients in a French-Canadian chiropractic teaching program: a mixed method study
Source: Chiropr Man Therap. 2025 Oct 27;33:50. doi: 10.1186/s12998-025-00611-1 (PMC12560557; doi:10.1186/s12998-025-00611-1)
Supplement: Supplementary file 1 — Supplementary Material 1 [file 12998_2025_611_MOESM1_ESM.docx]

**Appendix 1**

**TABLE OF CONTENTS**

Table 1. Association between PAM level of activation, body pain sites and chronicity (n= 190)

Table 2a: Practice style questionnaire mean scores for interns and clinicians

Table 2b: Interns and clinicians practice styles

Table 3: Patient’s interviews

Table 4. Intern’s interviews

Table 5. Clinician’s interviews

Table 1. Association between PAM level of activation, body pain sites and chronicity (n= 190)

| Factor | β Estimate | 95% CI | P-value |
| --- | --- | --- | --- |
| Cervical pain | -0.441 | -1.005 – 0.123 | 0.125 |
| Thoracic pain | -0.726 | -1.353 – -0.099 | 0.023* |
| Lumbar pain | -0.136 | -0.819 – 0.548 | 0.697 |
| Chronicity | -0.066 | -0.668 – 0.536 | 0.830 |

*p<0.05

Table 2a: Practice style questionnaire mean scores for interns and clinicians

| Practice style traits | Mean scores (SD) | | |
| --- | --- | --- | --- |
|  | Evidence-experience | Non conformity | Practicality |
| Interns | 19.9 (2.4) | 16.6 (3.0) | 14.8 (2.6) |
| Clinicians | 19.1 (2.2) | 17.1 (1.8) | 12.8 (4.8) |

SD=Standard deviation

Table 2b: Interns and clinicians practice styles

| Practice styles | Seekers  n (%) | Receptive  n (%) | Traditionalist  n (%) | Pragmatic  n (%) | Other  n (%) |
| --- | --- | --- | --- | --- | --- |
| Interns (n=38) | 8 (21%) | 6 (16%) | 2 (5%) | 21 (55%) | 1 (3%) |
| Cliniciens (n=26) | 1 (4%) | 10 (39%) | 4 (15%) | 11 (42%) | ----------- |

Table 3: Patient’s interviews

| Specific belief | Facilitators | Barriers | Participants Quotes | Themes |
| --- | --- | --- | --- | --- |
| Social influences (5 facilitators; 1 barrier) | Other professional’s influence, family’s influence, intern’s influence, coworkers, and being part of a group), | Family’s influence | "Let's say my partner tells me from experience that she did this and it works; that becomes something tangible for me, and I would probably use it." (P13)  "All the feedback I've received, both from my intern and from my family, is to continue with the exercises. So everyone is kind of encouraging me in that." (P1) | The intern is a positive influence. My surroundings are a positive influence. |
| Behavioral regulation (5 facilitators; 3 barriers) | The presence of pain serving as a reminder, visual reminders, accessing material, having a routine, and adapting quickly | Lack of attendance, not having visual clues, and having less pain. | "It's been about having my workout routine stable and then making sure that there are certain exercises that will really target the abdominal area because that's what it's all about, making sure that I'm adding to it." (P1)  "[The use of SMS] depends on the level of pain. Let’s say that when I have morning pain that prevents me from functioning normally, I tend to do some light stretches and warm-ups, because once my muscles are warmed up, my pain generally decreases by at least 75%" (P7) | Pain is a useful regulator in the monitoring of SMS, visual reminders are helpful. Patients missing their appointments |
| Emotions (5 facilitators; 6 barriers) | Feeling stressed or frustrated about not following SMS, pride, well-being and positive thoughts, positive influence, and absence of stress associated with SMS | Guilt, shame, frustration, stress, discouragement, and apprehension. | ‘’[…] I want to do it because I know it will make me feel better.’’ (P12)  ‘’It's a small source of stress or frustration when I don't do it.’’ (P12) | A sense of well-being provided by SMS, pride comes from performing SMS, SMS can be a source of guilt, stress, and frustration |
| Goals (13 facilitators, 0 barrier) | Routine integration, attendance, decreasing pain, better posture, responsibility, maintain health, reduce frequency of treatments, strengthening, freedom, avoiding medication, comfort and preventing pain. |  | ‘’ I've tried to include [SMS] in my routine in a simple way so that it doesn't just add another task to my routine.’’ (P12)  "I decided to take care of myself. That's why self-management is important to me, so that I can work on myself. " (P5) | Decreasing and preventing pain, improving physical characteristics, and taking responsibility for one's own care |

Table 4. Intern’s interviews

| Specific belief | Facilitators | Barriers | Participants Quotes | Themes |
| --- | --- | --- | --- | --- |
| Knowledge (6 facilitators; 6 barriers) | Knowing different SMS, being able to apply SMS after training, and knowledge about effectiveness of SMS | Lack of knowledge about SMS learned in the chiropractic program at UQTR, lack of knowledge about how to apply SMS and lack of knowledge about other health professionals. | "I personally don't remember having a full course on [brief action planning], but maybe we've learned so much that I don't remember, but it might be interesting to see it more in our program." (1I)  "No, it's the first time I [have heard of Brief Action Planning]. Other than using the Smart [approach] to apply ice or heat. [I find that knowledge on SMSS] are a bit unclear in the literature […]" (4I) | Variable levels of general knowledge, and how best to apply SMSS. |
| Environmental Context and Resources (4 facilitators; 7 barriers) | Attending extracurricular experience, knowledge shared by clinicians, use of databases to support use of SMS and the use of tools and documents available at UQTR outpatient clinic. | Absence of workshop on SMS, lack of time, clinicians lack of knowledge on SMS, lack of guidelines, clinicians lack of availability, the context of numerical requirements and the added workload are all barriers to the use of SMS | "There are many [clinicians] who have a lot of clinical experience and have been at the clinic for years, but who might be a bit less receptive to change. In my opinion, several clinicians may also not be the most up-to-date in terms of evidence." (9I) | Lack of a facilitated workshop on SMS, lack of consistency among clinicians regarding SMS, time and presence of good resources to assist interns in relation to SMS |
| Skills (4 facilitators; 6 barriers) | Interns have the skills, professionalism, interns have skills in providing stress management recommendations and interns with sports chiropractic have more tools related to SMS | Lack of skills, lack of experience and the inability to define and accompany patients in relation to their SMS | "[…] occasions when the patient told me about an objective, but we didn't define it properly and didn’t reach it. My lack of competence in implementing this kind of approach was probably the problem." (6I)  "I think the most important thing is really communication. It's essential to communicate with the patient and explain why [SMSS are] important." (2I) | Interns are able to guide their patients in regards to the use SMS, interns are not able to provide SMS and communication is an important skill to master for providing SMS |
| Memory, Attention and Decision Process (2 facilitators; 4 barriers) | determining the best moment to include SMS with the patient and modify SMS according to the patient | Dismissing use of SMS, lack of interest, lack of knowledge about the best moment to deliver SMS and waiting before talking about SMS. | "Over time, you develop a flair for saying "OK, this is the right moment for him to open the door for me, so I'm talking about [SMS]."  "I would suggest [exercises] to everyone. Of course, not at the first appointment, because it's very busy. " (6I) | Interns decide not to offer SMS at first appointment, interns do not offer SMS to reluctant patients and discussing with the patient the best time to offer SMS is a facilitator |
| Goals (5 facilitators; 1 barrier) | Desire to establish SMART goals, motivating patients, improving patient’s condition, importance of SMS and giving SMS to all patients. | Goal is not to give all the SMS at the first appointment | "I feel capable of giving [SMSS]. It's definitely something I want to do: providing self-management strategies." (10I)  "I find it important to incorporate the patient's wishes into my treatment plan. " (8I) | The goal of providing SMS from interns is a facilitator, interns want to educate patients on the importance of SMS and interns have a goal to do everything in their power to make their patients better. |

Table 5. Clinician’s interviews

| Specific belief | Facilitators | Barriers | Participants Quotes | Themes |
| --- | --- | --- | --- | --- |
| Knowledge (12 facilitators, 8 barriers) | : I read articles on health and SMS to enrich my knowledge, I refer to practice guides at 50%, I know Optima: a database for researchers, Gathering knowledge in a database facilitates SMS, I know the CCGI cervical exercises, I know that the literature supports self-management, I know that recommendations are adapted to the stage of the injury, I have learned that video exercises facilitate patient compliance, I know that motivational interviewing is more effective than recommendations from the practitioner, I am familiar with BAP, I've learned about SSAG through reading and conferences, I have learned that patients adhere more readily to written recommendations. | : I'm not familiar with SMS so I don't teach it, I don't recall having any training to guide the clinician or a student on how to approach a patient with SMS, our knowledge of personality types is limited so our strategies are probably not well adapted, I'm not familiar with BAP, training and evidence on BAPS would facilitate their use, I rarely consult articles on SMS, there is a lack of knowledge about SSAG, I didn't know about smart in the clinical context. | "Then again, since we don't have all the knowledge of the different personality types, our strategies probably always revolve around the same things." (8C)  "So, if there is training or resources put in place to help us [with SMSS], I think it will greatly help in the implementation. " (4C) | Training and access to scientific literature on SMSS would facilitate their use, senior interns have sufficient knowledge, clinician supervisors varying levels of knowledge and lack of training in SMSS are limiting factors, and written recommendations and audiovisual demonstrating exercises increase patients adherence. |
| Social Influences (7 facilitators, 6 barriers) | Social influence is positive; People who are important to me think I should offer SMS; People are always open to giving as much SMS as possible; Other professionals support the use of SMS by chiropractors; Intern-clinician interaction seems positive; The influence of SMS is positive, but there is still progress to be made as the concept of SMS is recent; It's a good idea to have positive models. | The clinic and its director should support the clinicians more | "At the same time, I think it gives patients, well, us, our profession, a certain credibility, because we want to do everything we can to help the person with their condition as much as possible. " (4C)  "definitely the relationship between the clinic director and the clinicians. is there frequent contact? is there good communication? Well, that's what should be important, for the clinicians to feel supported by the clinic, and for him to be a backer, to always feel that the clinic is behind you. " (5C) | Patients, interns, clinicians and other professionals are in favor of the use of SMS; The clinic and its director should support the clinicians more as SMSS is still a recent concept |
| Environmental context and resources (6 facilitators, 16 barriers) | Technological advances are available to monitor patient health data; There are sufficient resources to offer exercise-related CAMS; Chiropractors/chiropractic interns have a closeness to the patient that other healthcare professionals don't have; A clinical context where the exchange and sharing of knowledge in relation to SMS is advocated is a facilitator; The school curriculum enables interns to familiarize themselves with SMS and; Having several clinicians/supervisors facilitates the use of SMS. | There is a lack of clinical tools to facilitate the use of SMS; Lack of material with concise, structured information; Interns are not using the resources already available to them; Offering self-management support strategies requires more time; Having several clinicians/supervisors is a barrier to the use of SMS; Lack of training/guided workshops to offer more SMS; Administrative overload for interns is a barrier to the adoption of SMS; It is difficult to listen to podcasts and other audio content related to SMS; The relational context between clinicians is not conducive to the adoption of SMS; Pedagogical teaching periods (PEP) are not used to their full potential by clinicians to demonstrate SMS; Clinicians are not using the resources already available to them; There is a lack of technological advances and IT resources to offer SMS; There is a lack of resource persons/experts to guide interns in relation to SMS; Lack of manpower/clinicians is an obstacle to the adoption of SMS; It would be interesting to have forms to explain self-management; There is a lack of support for clinicians. | "I also believe that using different modes of presentation, i.e. above all a visual support, either pre-recorded vignettes or examples of helping relationships, will simply facilitate the integration of the ability to carry out these processes. " (2C)  "I think [interns] tend to have a lot on their shoulders, and it's often this aspect of the relationship with the patient that can suffer as a result." (2C) | Lack of time, manpower (low ratio of clinician to interns), clinical support tools (e.g., vignettes, algorithms), training/workshops, and resource persons; not all clinicians effectively use available resources. |
| Believes on Consequences (5 facilitators, 2 barriers) | A clinician who is convinced of the importance of SSAG will be more successful in proposing it to his patients; I believe that following SSAG will help the patient's condition; I believe that the patient will not recover completely without SSAG; There are no disadvantages to the use of SSAG in our patients and; SSAG promotes autonomy. | I doubt the effectiveness of SSAG in helping our patients and; I believe it is harmful for the patient not to follow my recommendations to the letter | "I think you always have to include the patient as much as possible in the treatment plan, and I think that in the long term that's what gives the best results too. " (4C)  "If you follow him and then guide him towards chronicity, it's like not giving him the tools to help himself and then knowing that the patterns will come back, the pain will come back. " (3C) | Clinicians believe that offering SSAG will help the patient in their condition, that the patient will not be able to heal completely without SSAG and that patients with SSAG will be more successful than those without SSAG is a facilitator and; SSAG is a way of empowering our patients. |
